# First Report of *Phaeoacremonium iranianum* Causing Olive Twig and Branch Dieback

**DOI:** 10.3390/plants11243578

**Published:** 2022-12-19

**Authors:** Elena Petrović, Karolina Vrandečić, Jasenka Ćosić, Gabriella Kanižai Šarić, Sara Godena

**Affiliations:** 1 Institute of Agriculture and Tourism, 52440 Poreč, Croatia; 2 Faculty of Agrobiotechnical Sciences Osijek, Josip Juraj Strossmayer University of Osijek, 31000 Osijek, Croatia

**Keywords:** *Phaeoacremonium iranianum*, olive, dieback

## Abstract

In an olive orchard on the western part of Istria, Croatia, twig and branch dieback was observed on several olive trees. In total, seven samples from symptomatic trees were collected. Samples were analyzed, and four fungal isolates showed morphological similarities to the species *Phaeoacremonium.* One isolate, chosen as a representative, was taken for molecular identification and pathogenicity tests. Based on the DNA sequence data of the ITS, TUB, and EF1α gene regions, the isolate was identified as *P. iranianum*. Pathogenicity tests were conducted on detached olive branches and olive trees in the greenhouse. To the best of our knowledge, this is the first report of twig and branch dieback on olive caused by *Phaeoacremonium iranianum*.

## 1. Introduction

Olive (*Olea europaea* L.) is one of the most important crops in the Mediterranean part of Croatia. According to the latest statistical data, the Croatian national production of olives is approximately 23,000 tones [1]. Olive trees are known to be drought-resistant and hardy but susceptible to several major diseases [2]. Recently, however, olive trees are becoming more susceptible to diseases caused by phytopathogenic fungi. We believe the main reasons for this increased susceptibility are changes in cultivation methods, the planting of infected plant material, increasing resistance of pathogens to fungicides, and climate extremes. In recent years, there have been various occurrences of new diseases in olive trees in Istria that were unknown even to experienced olive growers. Unfortunately, studies of pathogens associated with olive decline in Croatia are few. In order to create a plant protection strategy (within the framework of sustainable olive production) and for proper tillage of the soil before planting (especially if a crop that hosts the same diseases as olive was grown on the plot), the detection of the causal agents of these unusual olive diseases is crucial.

## 2. Materials and Methods

### 2.1. Sampling and Fungal Isolation

In 2021, olive trees which showed signs of twig and branch dieback, discoloration of the bark, and necrotic lesions were spotted in an olive orchard on the western side of Istria, Croatia. The area of the orchard was 0.43 ha and contained approximately 70 olive trees. Disease incidence was reaching 40%. Olive trees of the orchard (100% local cultivar ‘Buza’) were over 30 years old and grown on the soil where grapevine had been grown beforehand. In total, seven samples from seven trees (one sample per tree) of branches from symptomatic trees of ‘Buza’ were collected and brought to the laboratory for analysis. Small pieces of branches (4 × 4 mm) were rinsed under tap water, surface sterilized in 70% ethanol for one minute, rinsed two times in sterile distilled water, and placed on a sterile paper sheet in a laminar flow cabinet until dry. Pieces of branches were plated on PDA amended with 35 mg/L of penicillin and incubated. After five days of incubation at 25 °C under dark conditions, isolates were transferred onto the fresh PDA medium for pure culture.

### 2.2. Morphological and Molecular Identification

After 14 days of incubation at 25 °C in dark conditions, pure fungal cultures were taken for examination. Four isolates showed morphological similarities to the genus *Phaeoacremonium*. One isolate (R18 B4), chosen as representative, was taken for molecular identification. Total DNA from the isolate was extracted with Maxwell^®^ RSC Plant DNA Kit (Promega, Madison, WI, USA). The PCR reaction was performed using ITS1/ITS4 [3], Bt2a/Bt2b [4], and EF1-728F/EF1-986R [5] pair of primers. The PCR reaction mixture was composed of 12,5 µL of EmeraldAmp^®^ GT PCR Master Mix, 0,5 µL of each primer, 6,5 µL of nuclease-free water, and 5 µL of genomic DNA. Polymerase chain reactions (PCR) (Table S1) were conducted in a SureCycler 8800 Thermal Cycler (Agilent Technologies, Santa Clara, CA, USA). The PCR products were visualized on 1% agarose gel light using an iBright CL1000 Imaging System (Invitrogen, Thermo Fisher Scientific, Waltham, MA, USA). Purification of PCR products was conducted with the GenElute™ PCR Clean-Up Kit (Sigma-Aldrich^®^, Burlington, MA, USA), and sequencing (with EZ-Seq) of the PCR products was performed by Macrogen Europe (Amsterdam, The Netherlands). Sequences were edited in Sequencher^®^ (Gene Codes Corporation, Ann Arbor, MI, USA) and compared with sequences from GenBank^®^. Phylogenetic analysis (Figure S4) was performed using ITS sequence data from reference isolate R18 B4 and ITS sequence data of isolates (of species *P. iranianum*, *P. minimum*, and *Botryosphaeria dothidea*) using sequence data from GenBank. The sequences were aligned using ClustalX2 (UCD Dublin, Ireland) software, and a phylogenetic tree was made using MEGA11 (Pennsylvania State University, State College, PA, USA) software. Sequence alignment was generated from neighbor-joining tree.

### 2.3. Pathogenicity Tests of Isolate

Two pathogenicity tests were conducted to determine pathogenicity of the isolate on the olive tree: one on detached branches from cultivars ‘Buza’ and ‘Rosinjola’ in the laboratory and another one on the four-year-old olive tree of the cultivar ‘Rosinjola’ in the greenhouse. Detached branches were washed with water, surface sterilized in 10% sodium hypochlorite solution for 10 min, rinsed with sterile distilled water for 10 min, and placed in a laminar flow cabinet, on sterile paper, until dry. Branches were inoculated by placing a 4 mm-diameter mycelium plug from a 14-day-old PDA culture of R18 B4 isolate in a wound made with a 4 mm-diameter cork-borer. Wounds were sealed with Vaseline and protected with Parafilm. Ten branches in total, per cultivar, were used. Fungal treatments were compared to the control treatment inoculated only with PDA plugs without mycelia, sealed with Vaseline, and protected with Parafilm. Inoculated branches were kept in laboratory conditions for 20 days.

Branches from olive trees found in the greenhouse were chosen at random and inoculated the same way as previously described for detached branches. Inoculated plants had been kept in a greenhouse, at approximately 25 °C, for three months, from March to July 2022, and were monitored for the presence of symptoms. After incubation, samples were collected, and in an attempt to fulfill Koch’s postulate, small pieces of necrotic tissue from the edge of each lesion were cut and placed on PDA to recover inoculated fungus. 

## 3. Results

### 3.1. Sampling and Fungal Isolation

**Figure 1:**
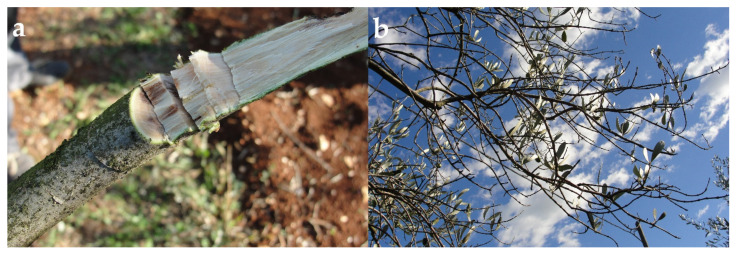
(**a**,**b**) Disease symptoms on olive branches in an orchard near Rovinj in Istria, Croatia, in the year 2021.

In the field, the symptoms of the disease on ‘Buza’ olive trees were the wilting and dieback of twigs and branches, as well as brown internal necrosis (Figure 1). Symptoms such as dieback were observed on lateral branches, on one side of the trees. When the outer layer of bark from the branches was scraped away, it was revealed that the brownish discoloration had extended on the surrounding tissue. Successful fungal isolation was obtained in four out of seven samples (57.1%). Isolations with saprophytic fungus were discharged.

### 3.2. Morphological and Molecular Identification

**Figure 2:**
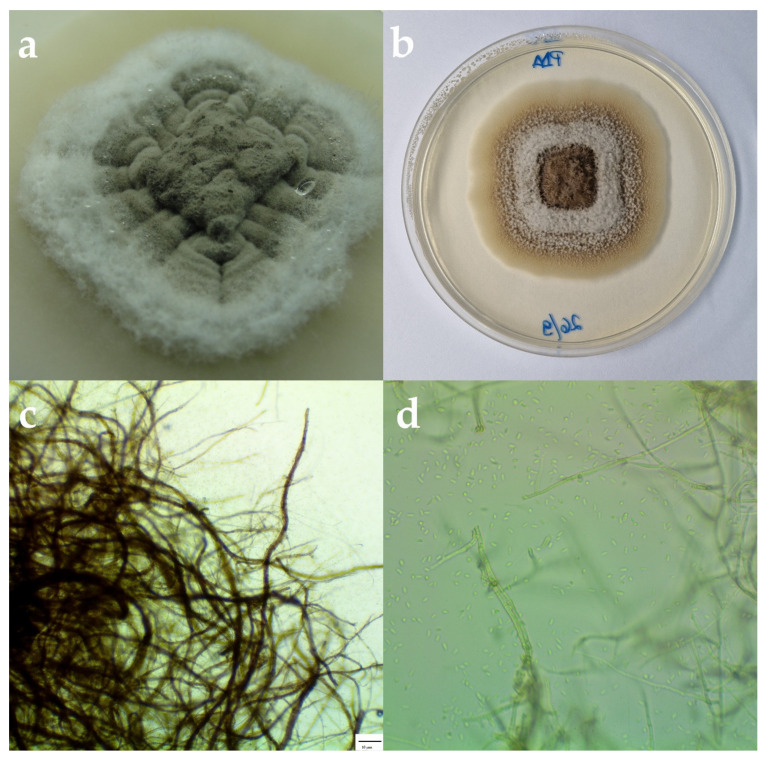
(**a**) *Phaeoacremonium iranianum* colony, on PDA, after two weeks in the dark at 25 °C. (**b**) *P. iranianum* colony, on PDA, after one month. (**c**) Micrographs of *P. iranianum* isolate under the microscope. Scale bar = 10 µm. (**d**) Hyaline, ovoid conidia.

Fungal isolates have been identified based on the colony characteristics (color, form, margin, elevation, surface, and opacity) and spore characteristics (color, presence or absence of septum, and shape). The developed fungal colonies were brownish on PDA, reverse darker brown; circular shaped with an entire edge; and with aerial, opaque, and cottony mycelium and branched septate hyphae (Figure 2). The isolate produced hyaline, unseptate, and ovoid conidia. An average conidia body length was (oblong-ellipsoidal) 4.5 × 1.5 µm. These morphological characteristics identified the fungus as *Phaeoacremonium iranianum* L. Mostert, Gräfenhan, W. Gams & Crous, 2006 [6]. For molecular identification, consensus sequences of representative R18 B4 isolate were produced (GenBank accession numbers: OP627795 for ITS, OP684932 for TUB, and OP684933 for the EF1α gene). BLAST analysis of the sequences showed 100% similarity with *P. iranianum* (reference number MG745842 for ITS, KF179086 for TUB, and KF764625 for the EF1α gene).

### 3.3. Pathogenicity Tests of Isolate

**Figure 3:**
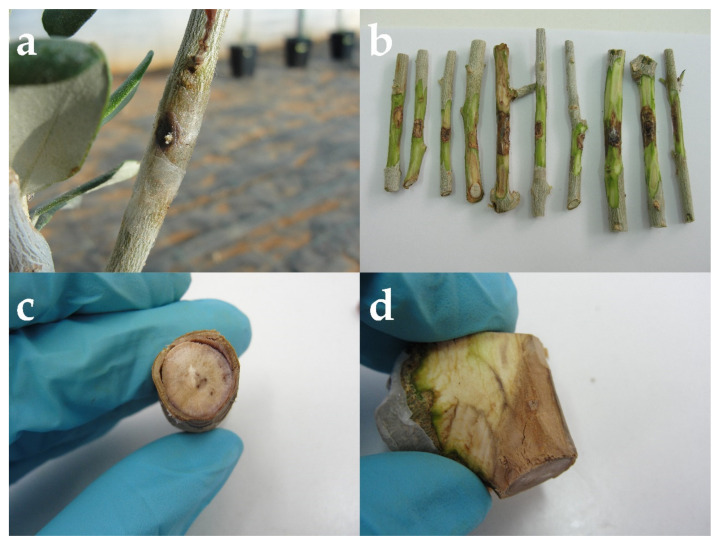
(**a**–**d**) Disease symptoms on branches used in pathogenicity tests from March to July 2022.

The symptoms of the disease on the olive branches tested in the laboratory and on the branches collected from olives in the greenhouse showed similar symptoms to the branch samples collected from the field survey. Brown streaking in cross-sections was detected (Figure 3), and when the outer layer of bark from the branches was scraped away, brown discoloration (staining) had extended around the affected tissue. The pathogen had been consistently reisolated from affected pieces of wood, while the controls remained healthy. To fulfill Koch’s postulate, one isolate chosen as a representative was carried out for molecular identification, as previously described, using an ITS5/ITS4 [7] pair of primers. Purification and sequencing of the PCR products were performed by Macrogen Europe (Amsterdam, Netherlands). BLAST analysis of the ITS5 and ITS4 sequences showed 100% similarity with *P. iranianum* (reference number MG745842).

## 4. Discussion

There are several species from the *Phaeoacremonium* genus associated with olive diseases worldwide: *Phaeoacremonium africanum* [8,9], *P. alvesii* [10], *P. italicum* [10,11,12], *P. minimum* [8,9,10,11,12,13,14], *P. oleae* [8,9], *P. parasiticum* [8,9,10,11,15], *P. prunicola* [8,9], *P. scolytii* [8,9,10,11,12], *P. spadicum* [8,9], and *P. sicilianum* [10,11]. *P. iranianum* is a species from the family *Togniniaceae* [6]. It was previously described as a plant pathogen on several species of woody plants, including almond trees [16,17], citrus trees [18], cypress trees [19], forest trees [20], grapevine [20,21,22,23,24,25,26,27], pome fruit (apple, quince, hawthorn, pear) [28], and prunus trees [29]. It has most commonly been reported as associated with Petri and Esca diseases, one of the most destructive declining diseases affecting grapevine [22]. As the observed infected olive trees were grown on the ground where grapevines were previously grown, and since olive and grapevine share common pathogens, such as phytopathogenic fungi from the *Botryosphaeriaceae* family, there is a high risk of transmission of *P. iranianum* between grapevines and olive trees [26]. Aerial spores can be dispersed between vineyards that are near each other and those established in close proximity to fruit orchards, ornamental trees, or numerous other woody hosts [26]. This poses a danger to olive trees, especially in the Mediterranean part of Croatia, where vines and olives are often grown together.

Before planting, it is imperative to correctly prepare the soil and to start with the healthiest planting material possible. Baloyi et al. [24] states pruning wounds on grapevine as entry sites for infection by *Phaeoacremonium* species, so it can be said that pruning wounds on olive can act as entry sites for infection, too. Measures such as pruning wound protection (this includes the use of chemicals with different modes of activity) and the disinfection of tools are necessary. Pruning should be carried out during dry weather, as spores are released during the rain. It is necessary to monitor olive orchards, remove infected parts of the tree, and remove them from orchards, as they can be a source of inoculum.

Since there is no data on protection strategies for species of *P. iranianum*, the need for further research (especially of research based on alternative nonchemical or biological control strategies that will enable growers to minimize chemical inputs, within the framework of sustainable olive production) is emphasized.

To the best of our knowledge, this is the first report of *Phaeoacremonium iranianum* causing olive twig and branch dieback on olive trees.

## Data Availability

All sequence data for isolate R18 B4 are available in NCBI GenBank following the accession numbers in the manuscript. Sequence data from Koch’s postulate, PCR amplification program, and phylogenetic tree are available in the Supplementary Materials.

## Supplementary Materials

The following supporting information can be downloaded at: https://www.mdpi.com/article/10.3390/plants11243578/s1. ITS4 and ITS5 sequences from Koch’s postulate. Table S1: PCR amplification program set according to Alves et al. (2006) [30]. Figure S1: Phylogenetic tree based on internal transcribed spacer (ITS) sequences alignment. The evolutionary history was inferred using the Neighbor-Joining method [31]. The optimal tree is shown. The evolutionary distances were computed using the Maximum Composite Likelihood method [32] and are in the units of the number of base substitutions per site. This analysis involved six nucleotide sequences. All ambiguous positions were removed for each sequence pair (pairwise deletion option). There was a total of 596 positions in the final dataset. Evolutionary analyses were conducted in MEGA11 [33].
