# Supplementary material for: First Report of Phaeoacremonium iranianum Causing Olive Twig and Branch Dieback
Source: Plants (Basel). 2022 Dec 19;11(24):3578. doi: 10.3390/plants11243578 (PMC9785478; doi:10.3390/plants11243578)
Supplement: Supplementary file 1 [file plants-11-03578-s001.zip › Figure S1.pdf]

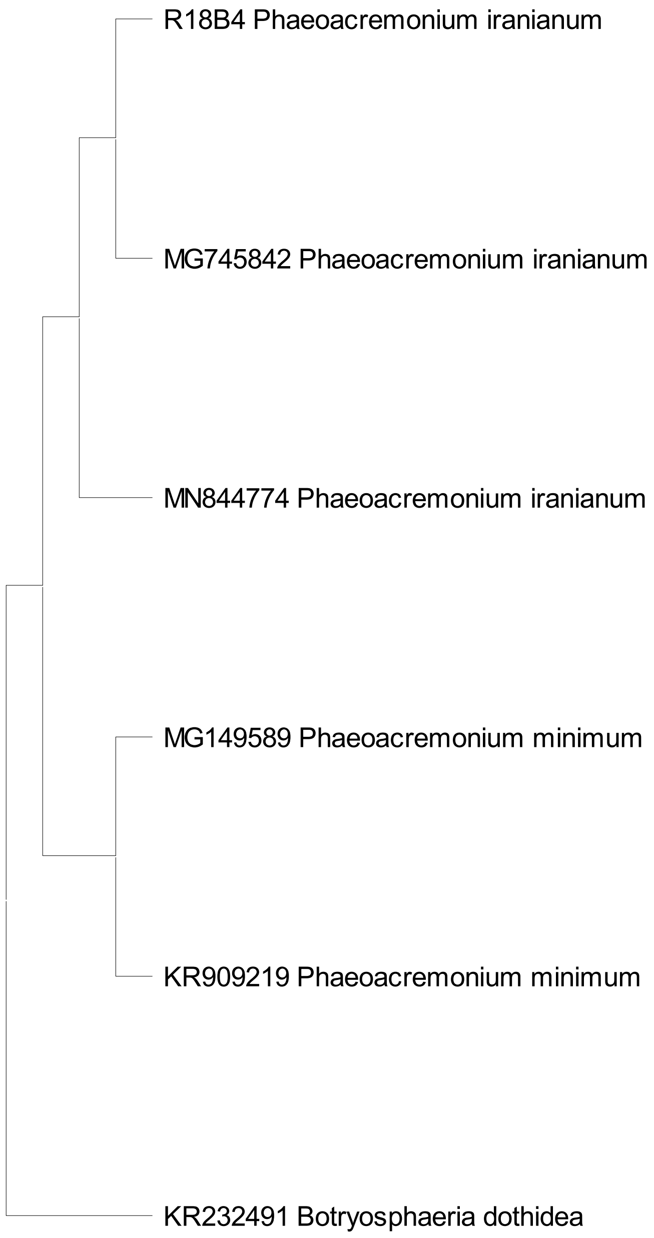

Figure S1. Phylogenetic tree based on internal transcribed spacer (ITS) sequences alignment. The evolutionary history was inferred using the Neighbor-Joining method [31]. The optimal tree is shown. The evolutionary distances were computed using the Maximum Composite Likelihood method [32] and are in the units of the number of base substitutions per site. This analysis involved 6 nucleotide sequences. All ambiguous positions were removed for each sequence pair (pairwise deletion option). There was a total of 596 positions in the final dataset. Evolutionary analyses were conducted in MEGA11 [33].
