# Supplementary material for: First Report of Phaeoacremonium iranianum Causing Olive Twig and Branch Dieback
Source: Plants (Basel). 2022 Dec 19;11(24):3578. doi: 10.3390/plants11243578 (PMC9785478; doi:10.3390/plants11243578)
Supplement: Supplementary file 1 [file plants-11-03578-s001.zip › Table S1.pdf]

Table S1. PCR amplification program set according to Alves et al. (2006) [30].

Hot Start 95 °C	Start Cycle 30 times	Denaturation 94 °C	Annealing 55 °C	Elongation 72 °C	End Cycle	Elongation 72 °C
**5 minutes**		30 seconds	45 seconds	1 minute and 30 seconds		10 minutes
